# Adverse events following immunization with typhoid conjugate vaccine in an outbreak setting in Hyderabad, Pakistan

**DOI:** 10.1016/j.vaccine.2020.03.028

**Published:** 2020-04-23

**Authors:** Farah Naz Qamar, Mohammad Tahir Yousafzai, Asif Khaliq, Sultan Karim, Hina memon, Amber Junejo, Inayat Baig, Najeeb Rahman, Shafqat Bhurgry, Hina Afroz, Uzma Sami

**Affiliations:** a Dept. of Pediatrics and Child Health, Aga Khan University Karachi, Pakistan; b Kirby Institute, University of New South Wales, Sydney, Australia; c Dept. of Pediatrics, Taluka Hospital Qasimabad, Pakistan; d Shah Bhitai Hospital, Latifabad, Pakistan

**Keywords:** Adverse events, AEFI, Typbar-TCV, Salmonella Typhi, Campaign, Vaccination, AEFI, adverse events following immunization, XDR, extensively drug resistant typhoid, TCV, typhoid conjugate vaccine, S.Typhi, salmonella Typhi, WHO, World Health Organization, EPI, expanded program on immunization, GAVI, Global Alliance for Vaccine and Immunization, IV, intravenous, ERC, ethical review committee, NBC, National Bioethics committee, Vi-DT, diphtheria toxoid conjugated vi-polysaccharide vaccine, ViCPS, Vi capsular polysaccharide, DG health, Director General Health, DC, Deputy commissioner, SEAP, Surveillance of Enteric Fever in Asia Project, MDR, Multi Drug Resistant

## Abstract

Pakistan is facing the world's largest outbreak of extensively drug-resistant (XDR) Typhoid. Vaccination campaign for children aged 6 months to 10 years old with Typhoid Conjugate Vaccine (Typbar-TCV®) was conducted in high-risk areas of Hyderabad during 2018. About 207,000 children were vaccinated. Here we report the adverse events following immunization (AEFI) during the campaign. The campaign was carried out using outreach and fixed centre strategy. Community mobilizers visited each household to perform line listing and mobilize parents with age-eligible children. Children were observed for 30 min post-vaccination. Two-pronged strategy was used for ascertainment of AEFI. A 24/7 hotline number was provided to all parents/caretakers (n = 199,861) to report AEFI during 14 days following immunization. An age-stratified (n = 7139 children) were actively followed at days 7 and 14 for the ascertainment of AEFI. All AEFI were examined by three trained medical officers. A structured questionnaire using Brighton collaboration criteria with level 3 diagnostic certainty was used for the recording of AEFI. Data were analysed using Microsoft Excel Office 365. Overall, 499 AEFI (433 in the subset actively followed and 66 self-reported through hotline) were observed. The rate of AEFI was significantly higher among very young children (age group 6 to 12 months) as compared to 2 to 3 years old children (0.54% vs. 0.33% respectively; p-value < 0.001). Fever was the most common AEFI self-reported through the hotline (38/199,861 = 0.02%) and among the subset followed actively for 14 days (206/7139 = 2.89%). Fever was followed by local reactogenicity 10/199,861(0.01%), and 134/7139 (1.88%) through self-reported hotline and active follow-up, respectively. No serious AEFI was observed. Administration of a single dose of Typbar-TCV among children aged 6 months to 10 years old during an outbreak setting of Hyderabad Pakistan was safe.

## Highlights

• Post licensure safety data of Typbar-TCV® is not available from a population wide campaign setting.
• 207,000 doses of Typbar-TCV were administered in a campaign setting of Hyderabad, Pakistan.
• No death, disability or hospitalization associated with Typbar-TCV during 14 days of vaccination was observed.
• Fever and local reactogenicity were the frequently observed adverse events.

## 1 Introduction

Typhoid is a communicable disease caused by *Salmonella* enterica serovar Typhi (*S.* Typhi). The disease is characterized by fever, vomiting, and diarrhea and is highly endemic among the south-east Asian and African regions [1], [2]. According to the global burden of disease estimates, 10.9 million cases and 116.8 thousand deaths due to *S*. Typhi occurred during 2017. South Asia was the most affected region with typhoid accounting for 72% of the global cases and 70% of the deaths [3]. Morbidity and mortality were highest among children 5–9 years of age (56% of cases and 59% of deaths) followed by children less than 5 years old (12.6% of cases and 17% of deaths) respectively [3]. While globally the burden of *S.* Typhi and its associated mortality has declined since 1990, increasing resistance against antimicrobial resistance in *S.* Typhi remained a global public health threat [3], [4], [5].

Pakistan situated in South Asia has one of the highest burdens of typhoid fever in the region [*unpublished SEAP study data*]. In Pakistan, more than 90% and 50% of *S.* Typhi are resistant to fluoroquinolone and multidrug-resistant (MDR = resistant to ampicillin, trimethoprim-sulfamethoxazole, chloramphenicol) respectively [6]. In November 2016, an outbreak of extensively drug-resistant (XDR = resistant to ampicillin, chloramphenicol, trimethoprim-sulfamethoxazole, fluoroquinolones, and 3rd generation cephalosporin) *S.* Typhi occurred in Hyderabad city of Sindh, Pakistan. The outbreak started in two sub-districts of Hyderabad and subsequently spread to all of Hyderabad and other adjacent cities in Pakistan. More than 5000 cases of XDR *S.* Typhi have been reported so far. About more than 90% of the XDR typhoid cases are children younger than 15 years of age and about 70% of the cases are below 10 years old [7], [8], [9]. The outbreak of XDR *S.* Typhi in Hyderabad significantly reduced available treatment options resulted in a higher number of hospitalizations and cost of treatment. In this scenario, mass immunization with typhoid conjugate vaccine (TCV) seemed like a rapid and most appropriate measure to contain the outbreak [10].

As part of the emergency response, the Aga Khan University Karachi in collaboration with the ministry of health Sindh initiated a mass immunization campaign with typhoid conjugate vaccine (Typbar-TCV® manufactured by Bharat Biotech International Limited) targeting children aged 6 months to 10 years old. Typbar-TCV® containing polysaccharide of *S.* Typhi Ty2 conjugated to Tetanus Toxoid is the only TCV vaccine prequalified by the World Health Organization (WHO) [11], [12]. Although, Typbar-TCV® has been reported to be highly effective and safe among children aged 6 months and above, its safety during population-wide campaign setting is not yet established [13], [14], [15]. Therefore, this is the first population-wide safety data from the campaign setting on Typbar-TCV® which provides important evidence for the policymakers and health practitioners in the developing world where typhoid is still endemic. The data has important implications for GAVI, the vaccine alliance and GAVI eligible countries to introduce TCV in their expanded program on immunization (EPI). Herein we report the adverse events following immunization (AEFI) with Typbar-TCV® among children aged 6 months to 10 years old in an outbreak setting of Hyderabad, Pakistan.

## 2 Material and Methods

### 2.1 Study setting and XDR typhoid outbreak

Hyderabad is the second largest city of Sindh Province in Pakistan with a population of about 2.2 million [16]. The city is situated on the east bank of the Indus river about 150 km away from Karachi. Most of the population in Hyderabad are Sindhi and Urdu speaking. Hyderabad city is divided into four administrative units named Qasimabad, Latifabad, Hyderabad urban and Hyderabad rural. Qasimabad and Latifabad were the two adjacent areas where the outbreak of XDR typhoid started in November 2016 and spread to the rest of the country.

### 2.2 Vaccination campaign

In February 2018, The Aga Khan University Karachi in collaboration with the Ministry of Health Sindh initiated a community-based mass immunization campaign with Typbar-TCV® in the two affected areas of the Hyderabad city in Sindh, Pakistan. Temporary outreach vaccination centers were established in health houses (house of a lady health worker), private general practitioners' clinics, schools, *madrassah* (institutions where Islamic education is provided), *awtaaq* (guest room within house), and where no place was found, a campaign vehicle (Hi-Roof Bolan) was used as a vaccination camp. Female community mobilizers from the same community visited every household for a line-listing of the household, identification of target age children, and motivating parents to bring their children to the temporary vaccination center for free typhoid vaccination. There were 18 vaccination teams and each team was composed of 2 community mobilizers, 1 vaccinator and 1 team leader for screening and data collection. Three trained medical officers and a research supervisor were hired to supervise the teams, observe any AEFI, manage AEFI and provide a referral to the nearest hospital.

### 2.3 Target population and eligibility criteria

Children aged 6 months to 10 years old, living in the affected community of Latifababad and Qasimabad, who received no typhoid vaccine (reported by their parent) during the last one year and whose parents provide written informed consent for vaccination were eligible to receive a single dose of Typbar-TCV in this campaign. Children with an acute illness at the time of vaccination or allergy to any vaccines were excluded.

### 2.4 Vaccine, dose, and route of administration

We used five-dose Typbar-TCV ® vials in this campaign. A single 0.5 ml dose of Typbar TCV was composed of 25 µg of antigen + 4.5 mg of sodium chloride + 5 mg of 2-Phenoxyethanol preservative in 0.5 ml water for injection. The injection was administered with 1 ml auto-disable syringe either in the lateral thigh (if age was 6 months to 2 years) or deltoid muscle (if age was 2–10 years).

### 2.5 Training of staff

All vaccinators were trained for three days before the initiation of the vaccination campaign. The training was composed of correct administration of intramuscular injection, preparation of correct dose, safe disposal of sharps, prevention of needle stick injuries, cold chain management, log sheet for recording daily temperature, daily vaccine inventory sheet, aseptic technique for prevention of infection during injection administration and identifying any immediate AEFI, line of action/communication and use of emergency kits in case of anaphylaxis. The emergency kits containing essential lifesaving medicine, cannula, disposable syringe, IV infusion set, and IV fluids were provided to each vaccination team. All team leaders and vaccinators were advised to counsel the parents/caretakers to wait for 30 min post-immunization and report immediately to the teams if there is swelling, redness, respiratory difficulty, vomiting, diarrhea, fever, seizure, etc. Besides, the teams were also trained to counsel parents regarding the 24/7 hotline number and call on the number in case of an AEFI.

The three research medical officers were further trained for the management of adverse events, use of emergency kits for managing emergencies, referrals, interviewing skills, assessment to ascertain AEFI for recording on the AEFI tool using the Brighton collaboration criteria 2005 [17]. There were two tertiary care hospitals located in Qasimabad and Latifabad which were kept ready for management of any serious adverse events.

### 2.6 Ascertainment of AEFI

We used level 3 Brighton criteria for the ascertainment of AEFI. Level 3 definitions have the lowest level of diagnostic certainty however, the highest level of sensitivity [17]. These definitions are suitable for post-marketing surveillance in a resource-poor setting like our setting. AEFIs were identified using two strategies:

(1) Self-reported AEFI through 24/7 hotline: All children were observed for 30 min post-vaccination. Besides, all parents of the vaccinated children were provided a vaccination card with a printed hotline number. Parents were counseled by the team leaders to immediately call on the given number if their child develops any illness during 14 days of post-vaccination. Parents were also assured that the medical officer will visit the child and the necessary referral will be provided in case of any emergency. The research supervisor monitors this hotline for 24/7 and a standby vehicle with driver and research medical officer immediately responded to any AEFI reported through hotline. The medical officer after assessing the AEFI provided necessary treatment or referral to the nearest hospital. Information on the AEFI was recorded in the structured data collection tool.
(2) Active surveillance for AEFI: A subset of the parents/caretakers of age-stratified n = 7139 vaccinated children (6 month-1 year, 1–2, 2–3, 3–4, 4–5 and 5–10 years) were selected using systematic random sampling (every 10th vaccinated child from each of the age stratum). Enrolment in active surveillance for AEFI was stopped once the given sample size was achieved. If the parents refused to be followed on days 7 and 14 by the study team, the next age-eligible child was enrolled. Parents of the children enrolled in the active surveillance were provided the vaccination cards with a 24/7 hotline number for emergency contact and weekly diary in the local language (Urdu/Sindhi) for daily recording of the health condition of the child. The English version of the weekly diary is attached in Appendix A. Research medical officers visited these households on days 7 and 14 following the immunization to collect the weekly diaries, interview parents and ascertain any AEFI for recording in the structured AEFI tool. If the child was ill during the previous 7 days and visited any hospital or clinic and not being reported to the vaccination team, the research medical officer tried to collect information regarding any tests done, prescriptions, diagnoses made by the consulting physician.

### 2.7 Data collection tool

A structured questionnaire was prepared in English and translated into the Urdu language and back translated into English to check for consistency. Trained research medical officers used the tool for recording all AEFI. The questionnaire was composed of information regarding the date of interview, batch number of the vaccine vial, expiry date, enrolment number of the child, age, gender and date of vaccination, date of onset of AEFI, description of AEFI, duration of AEFI, management of AEFI, and outcome, etc.

AEFI were broadly categorized as serious and non-serious AEFI. A serious AEFI was one in which a child following vaccine administration suffered from any life-threatening illness, i.e., convulsion, anaphylactic reaction, difficulty breathing, or any unexpected illness that required immediate medical attention, or hospitalization and this may lead to disability or death. Symptoms such as fever, nausea & vomiting, swelling, redness, itching, pain at the injection site, etc. predominantly self-limiting were considered as non-serious AEFI. The non-serious AEFI were further classified as mild (need no medical intervention), moderate (need medical intervention without affecting daily life activities) and severe (need immediate medical care and may result in complications, hospitalization, and death if left untreated) [18].

#### 2.8 Statistical analysis

All data were entered into the database prepared using Microsoft Excel office 365 and cleaned before analysis. Only descriptive analysis was performed. The frequency with percentage for total AEFI and types of AEFI was calculated. Besides, the percentage of AEFI among children of different age groups were also calculated. Chi-square test for trend was used to calculate the p-value for change in percentage of AEFI in the different age groups.

#### 2.9 Ethical consideration

Ethical approvals were obtained from the ethical review committee (ERC) of Aga Khan University Karachi and the national bioethics Committee (NBC) of Pakistan. Written informed consent was obtained from either of the parent or caretakers of the children before the vaccination.

## 3 Results

**Fig. 1:**
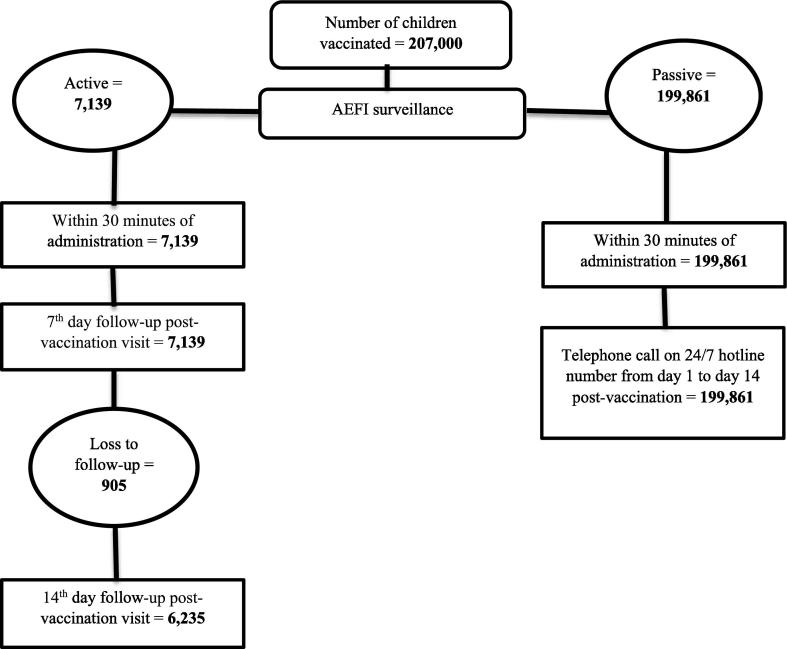
TCV vaccination and follow-up among vaccinated children.

A total of 207,000 children living in the high-risk areas of Hyderabad were vaccinated with Typbar-TCV. Among the vaccinated children, 106,522 (51.5%) were male. A slightly higher proportion of children (260,82/207,000 = 12.6%) were vaccinated in the age group 3–4 years while only 7% (n = 144,90) of the children aged 6–12 months were vaccinated (Table1). Out of the total, 7139 (3.4%) children of different age groups were actively followed-up at day 7 and 6235(3.0%) children were followed-up at both days 7 and 14 (n = 905 were lost to follow-up) (Fig. 1).

**Table 1:** Age and gender distribution of the vaccinated children.

Variable	Total children vaccinated N=207,000(%)	Random sample for active follow-up N=7139(%)
Gender:		
Male	106522 (51.5)	3719(52%)
Female	100478 (48.5)	3420 (48%)

Age group of vaccinated children		
6–12 months	14490 (7.0)	1107 (15.5%)
13–24 months	23805 (11.5)	1242 (17.4%)
25–36 months	26082 (12.6)	1285 (18%)
37–48 months	25875 (12.5)	1278 (17.9%)
49–60 months	25668 (12.4)	1142 (16%)
61–72 months	19665 (9.5)	214 (3%)
73–84 months	19872 (9.6)	250 (3.5%)
85–96 months	18216 (8.8)	228 (3.2%)
97–108 months	14076 (6.8)	193 (2.7%)
109–120 months	19251 (9.3)	200 (2.8%)

### 3.1 Total AEFI (N = 207,000)

**Fig. 2:**
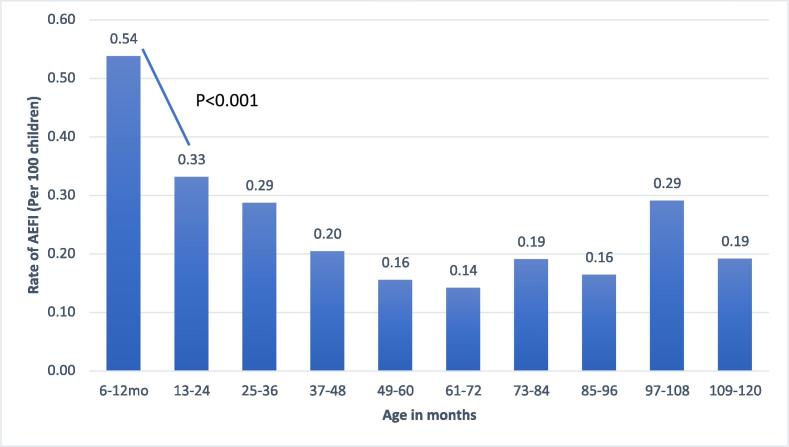
Age stratified AEFI rates reported through active follow up or self-reported by the parents though hotline.

There was no serious AEFI (hospitalization, death or disability) among vaccinated children during 14 days following immunization. Overall, there were 499/207,000 (0.24%) AEFI observed among the total vaccinated children. Fever was the most common AEFI (244/207,000 = 0.12%), followed by local reactogenicity (144/207,000 = 0.07%) and diarrhea (36/207,000 = 0.02%). Only one child suffered from syncope resulting in transient unconsciousness and one child started vomiting immediately after the vaccination. (Table 2). The rate of AEFI was significantly higher among very young children (age group 6 to 12 months) as compared to the 2 to 3 years old children (0.54% vs. 0.33% respectively; p-value <0.001). There was a consistent decreasing trend in AEFI till 6 years old children followed by an inconsistent slightly increasing and decreasing pattern of AEFI (Fig. 2). The rates of AEFI among male children were significantly higher as compared to female children (282/106,522 = 0.3% vs. 217/100,478 = 0.2%; p < 0.01).

**Table 2:** Distribution of adverse events self-reported during14 days post-immunization among total vaccinated children and subset of children followed-up for monitoring of adverse events.

Types of AEFI	Self-reported (N = 199861) n(%)	Monitored during follow-up (N = 7139) n(%)	Total (N = 207,000 n(%)
Fever	38(0.02)	206(2.89)	244(0.12)
Pain or swelling on injection site	10(0.01)	134(1.88)	144(0.07)
Diarrhea	4(<0.01)	32(0.45)	36(0.02)
Syncope	2(<0.01)	0	2(<0.01)
Local rash/itching	3(<0.01)	4(0.06)	7(<0.01)
Nausea & vomiting	3(<0.01)	11(0.15)	14(0.01)
Cold and cough	6(<0.01)	19(0.27)	25(0.01)
Any other	0	27(0.38)	27(0.01)

### 3.2 AEFI ascertained through active follow-up on days 7 and 14 (N = 7139)

There were 433/7139 (6.1%) AEFI ascertained through active follow-ups on days 7 and 14. Fever was the most common AEFI (206/7139 = 2.89%), followed by local reactogenicity (134/7139 = 1.9%) and diarrhea (32/7139 = 0.5%). (Table 2).

### 3.3 AEFI self-reported through a hotline (N = 199,861)

Only 66 (66/199,861 = 0.03%) of the AEFI were reported through the hotline. Similar to active follow-up, fever was the most frequently reported adverse event (38/199,861 = 0.02%), followed by local reactogenicity (10/199,861 = 0.01%) and cold/cough (6/199,861=<0.01%). (Table 2).

## 4 Discussion

We administered 207,000 doses of the Typbar-TCV® vaccine and only 499 adverse events were observed during 14 days post-immunization. About three-quarters of the adverse events were either fever or local swelling/pain on the injection site. Adverse events were highest among the youngest children which gradually decreases with increasing age. No serious adverse events resulting in hospitalization or deaths were observed.

Our finding of local reactogenicity (1.9%) and fever (2.9%) among the subset of vaccinated children who were actively followed-up is consistent with the pre-licensure phase 3 trial of Typbar-TCV® where the fever was identified among 4% and local reactogenicity among 3% of the children vaccinated with Typbar-TCV [14]. Similarly, a recently conducted phase IV trial of Typbar-TCV in Nepal which compared Typbar-TCV with AfroMenA vaccine randomized about 20,000 children aged 9 months to 16 years old in the two arms and actively assessed AEFI during 20 min post-immunization, 7th day of immunization, and then 3 monthly phone calls for the ascertainment of serious adverse events. The study reported 5% children with pain at injection site, 6% children experiencing unwell, 5.2% children with fever, 1.2% vomiting and 1.7% children with diarrhea in the TCV arm. The study also reported 1 serious adverse event potentially associated with the vaccine during 28 days following the immunization. There was no mortality attributed to TCV observed in the trial. There was no statistically significant difference in AEFI between the intervention and control arms [13].

Beside Typbar-TCV, our findings are also consistent with other types of typhoid conjugate vaccines which are still in the process of pre-qualification. A phase IV clinical trial using PedaTyph® (manufactured by Bio-Med India, containing Vi capsular polysaccharide of *Salmonella* typhi conjugated with tetanus toxoid protein) assessed the safety of the vaccine among 2200 children aged 6 months to 12 years old in Kolkata, India. Two doses of the vaccine were administered keeping a gap of 6 weeks in between and adverse events were observed for 30 min, 1 month and 1 year after vaccination. Pain (9%), redness (0.3%), swelling (3%) and fever (3.4%) were the most common adverse events observed within 30 min of the first dose of vaccine administration. No serious adverse events were observed either immediately or during one year after vaccination. Clinical events other than typhoid such as fever with cough and cold, tonsillitis, acute gastroenteritis, etc. during 1-year post-PedaTyph® vaccination were similar between the intervention and control arm [19]. Another study in Manila, Philippine evaluated the safety of diphtheria toxoid conjugated vi-polysaccharide vaccine (Vi-DT) in phase 1 trial among children aged 2 years and above reported local reactogenicity followed by fever as the most common adverse events among the vaccinees [20]. Similarly, a phase II trial conducted in the Philippine assessed the immunogenicity and safety of Vi-DT among children aged 6 months to 2 years old. The trial enrolled 76 children into the Vi-DT arm and children were observed for 60 min post-immunization, followed by 7th and 28 days of follow-up for the ascertainment of AEFI. Only one immediate reaction (erythema/redness and fever), mild in severity which resolved without sequelae was observed. The study also reported pain and tenderness and fever as the most common AEFI. There was no significant difference in AEFI rates between the children in Vi-DT arm versus placebo arm [21]. Like our findings, none of the trials reported any serious adverse events or adverse events resulting in sequelae.

Furthermore, our results are also consistent with the other types of typhoid vaccines such as Vi capsular polysaccharide (ViCPS) and Ty21a vaccines. According to the WHO global vaccine safety report, the rate of fever among children vaccinated with Ty21a ranges from 0.3 to 4.8% and 0–2% among children vaccinated with ViCPS [4].

Fever and local reactogenicity observed in this study were mild and self-limiting without any sequelae. Overall, the rate of adverse events was highest among the youngest age group (6–12 months) and lowest among children aged 5–6 years old. The rates of AEFI were inconsistent among children above 7 years of age. Pre-licensure trials on typhoid conjugate vaccines and other typhoid vaccines did not report any age-specific variations in AEFI. One possible explanation for the higher rates of AEFI among the youngest age group could be the result of parents being more caring for the infants and hence most likely reporting any mild illness or discomfort through the 24/7 hotline as compared to the older children.

While our AEFI estimates from the subset which was actively followed at days 7 and 14 is consistent with the earlier studies, the AEFI rate self-reported by the parents/caretakers through 24/7 hotline is extremely low. We did not find any study which assessed the safety of any TCV using this technique however, similarity of our findings from the active follow-ups with the other studies clearly demonstrate that self-reported technique for the assessment of vaccine safety underestimate AEFIs. There could be several reasons for the underestimation of self-reported AEFI nonetheless, the most plausible explanation seems to be the lower literacy rate among target population. According to the recent Pakistan demographic health survey, 24% of men and 34% of women in urban Sindh are illiterate [22]. This is also evident from the fact that none of the parents/caretakers in our subset population for the active follow-up completed the weekly diary for the daily recording of their children’ illness and complaints. Thus, self-reported AEFI by parents or caretakers using 24/7 hotline might prove to be the cost-effective strategy in educated population, however, in our setting it could not deliver the desirable results.

While this is the first study from the population-based campaign setting reporting post-licensure AEFI associated with Typbar-TCV® among children vaccinated in a campaign, several limitations need to be considered. Firstly, no control or comparison group of children was available to compare the rate of adverse events or measure the background risk of adverse events. Secondly, adverse events were only monitored for 14 days either through self-reported telephone calls or weekly follow-up visits. Thus, any distant adverse events occurring post 14 days of the vaccination could not be ascertained in this study. Thirdly, most of the families were uneducated and hence daily diary for the recording of any adverse events including fever following immunization could not be maintained. Therefore, research medical officers used to interview such parents and subjectively collect information on adverse events. While this might have underestimated some mild adverse events which the parents might have not recalled, the probability of missing any significant or serious adverse event is very unlikely. Fourthly, we were only able to follow-up a fraction of the total children vaccinated and hence any rare serious adverse event resulting in hospitalization, death, or disability among the rest of the children not being reported through the hotline is not impossible. Future studies with the hospital surveillance system for monitoring of AEFI with Typbar-TCV® especially for longer than 14 days following vaccination is recommended.

## 5 Conclusion

A single dose of Typbar-TCV® administered among children aged 6 months to 10 years old in campaign setting in Hyderabad city of Pakistan was safe. Fever, local reactogenicity, pain, and diarrhea were some of the common AEFI. No serious adverse events were reported. Mild fever as AEFI was significantly higher among very young children (6 months- 1 year) as compared to children older than 1 year.

## Declaration of Competing Interest

The authors declare that they have no known competing financial interests or personal relationships that could have appeared to influence the work reported in this paper.

## References

[1] Dewan A.M., Corner R., Hashizume M., Ongee E.E. (2013). Typhoid fever and its association with environmental factors in the Dhaka metropolitan area of Bangladesh: a spatial and time-series approach. PLoS Negl Trop Dis.

[2] Kim J.-H., Im J., Parajulee P., Holm M., Cruz Espinoza L.M., Poudyal N. (2019). A systematic review of typhoid fever occurrence in Africa. Clin Infect Dis..

[3] GBD 2017 Typhoid and Paratyphoid Collaborators. The global burden of typhoid and paratyphoid fevers: a systematic analysis for the Global Burden of Disease Study 2017. Lancet Infect Dis. 2019;19:369-81. 10.1016/S1473-3099(18)30685-6 PMC6437314 30792131

[4] World Health Organization. ANTIMICROBIAL RESISTANCE Global Report on Surveillance 2014.

[5] Browne A.J., Kashef Hamadani B.H., Kumaran E.A.P., Rao P., Longbottom J., Harriss E. (2020). Drug-resistant enteric fever worldwide, 1990 to 2018: a systematic review and meta-analysis. BMC Med..

[6] Qamar F.N., Yousafzai M.T., Sultana S., Baig A., Shakoor S., Hirani F. (2018). A retrospective study of laboratory-based enteric fever surveillance, Pakistan, 2012–2014. J Infect Dis..

[7] Klemm EJ, Shakoor S, Page AJ, Qamar FN, Judge K, Saeed DK, et al. Emergence of an Extensively Drug-Resistant Salmonella enterica Serovar Typhi Clone Harboring a Promiscuous Plasmid Encoding Resistance to Fluoroquinolones and Third-Generation Cephalosporins. mBio. 2018;9. 10.1128/mBio.00105-18 PMC5821095 29463654

[8] Yousafzai M.T., Qamar F.N., Shakoor S., Saleem K., Lohana H., Karim S. (2019). Ceftriaxone-resistant Salmonella Typhi Outbreak in Hyderabad City of Sindh, Pakistan: high time for the introduction of typhoid conjugate vaccine. Clin Infect Dis..

[9] Qamar F.N., Yousafzai M.T., Khalid M., Kazi A.M., Lohana H., Karim S. (2018). Outbreak investigation of ceftriaxone-resistant Salmonella enterica serotype Typhi and its risk factors among the general population in Hyderabad, Pakistan: a matched case-control study. Lancet Infect Dis.

[10] Andrews J.R., Qamar F.N., Charles R.C., Ryan E.T. (2018). Extensively drug-resistant typhoid - are conjugate vaccines arriving just in time?. N Engl J Med..

[11] World Health Organization. Typbar TCV® from Bharat Biotech, World’s First Typhoid Conjugate Vaccine Prequalified by WHO World Health Organization; 3rd January 2018.

[12] World Health Organization (2019). Typhoid vaccines: WHO position paper, March 2018 – Recommendations. Vaccine.

[13] Shakya M., Colin-Jones R., Theiss-Nyland K., Voysey M., Pant D., Smith N. (2019). Phase 3 efficacy analysis of a typhoid conjugate vaccine trial in Nepal. N Engl J Med..

[14] Mohan V.K., Varanasi V., Singh A., Pasetti M.F., Levine M.M., Venkatesan R. (2015). Safety and immunogenicity of a Vi polysaccharide-tetanus toxoid conjugate vaccine (Typbar-TCV) in healthy infants, children, and adults in typhoid endemic areas: a multicenter, 2-cohort, open-label, double-blind, randomized controlled phase 3 study. Clin Infect Dis.

[15] Jin C., Gibani M.M., Moore M., Juel H.B., Jones E., Meiring J. (2017). Efficacy and immunogenicity of a Vi-tetanus toxoid conjugate vaccine in the prevention of typhoid fever using a controlled human infection model of Salmonella Typhi: a randomised controlled, phase 2b trial. Lancet.

[16] Pakistan Bureau of Statistics. 6th Population and housing census - 2017. Pakistan2017.

[17] Kohl K.S., Bonhoeffer J., Braun M.M., Chen R.T., Duclos P., Heijbel H. (2005). The Brighton Collaboration: creating a global standard for case definitions (and guidelines) for adverse events following immunization. Advances in patient safety: from research to implementation (volume 2: concepts and methodology): Agency for. Healthcare Res Quality (US).

[18] Liu D., Wu W., Li K., Xu D., Ye J., Li L. (2015). Surveillance of adverse events following immunization in China: Past, present, and future. Vaccine.

[19] Mitra M., Shah N., Ghosh A., Chatterjee S., Kaur I., Bhattacharya N. (2016). Efficacy and safety of vi-tetanus toxoid conjugated typhoid vaccine (PedaTyph) in Indian children: School based cluster randomized study. Hum Vaccin Immunother.

[20] Capeding M.R., Teshome S., Saluja T., Syed K.A., Kim D.R., Park J.Y. (2018). Safety and immunogenicity of a Vi-DT typhoid conjugate vaccine: Phase I trial in Healthy Filipino adults and children. Vaccine.

[21] Capeding M.R., Alberto E., Sil A., Saluja T., Teshome S., Kim D.R. (2019). Immunogenicity, safety and reactogenicity of a Phase II trial of Vi-DT typhoid conjugate vaccine in healthy Filipino infants and toddlers: a preliminary report. Vaccine.

[22] National Institute of Population Studies (2019). Paksitan demographic and health survey 2017–18.

