# Supplementary material for: Adverse events following immunization with typhoid conjugate vaccine in an outbreak setting in Hyderabad, Pakistan
Source: Vaccine. 2020 Apr 23;38(19):3518–23. doi: 10.1016/j.vaccine.2020.03.028 (PMC7166079; doi:10.1016/j.vaccine.2020.03.028)
Supplement: Supplementary data 1 [file mmc1.docx]

**Appendices-I**

**Daily monitoring diary for typhoid conjugate vaccine AEFI**

| Participant ID number |  |
| --- | --- |
| Enrolment week |  |
| Child name |  |
| Child age (in months) |  |
| Child sex |  |
| Date of vaccination  ^(dd/mm/yyyy)^ |  |
| Supervisor contact number |  |

**Instructions:** You are requested to fill this dairy daily. If your child experiences any sign and symptoms mark (✓) otherwise keep it blank.

| Day (follow-up day) | Date  (dd/mm/yyyy) | Symptoms observed following administration of Typhoid conjugate vaccine | | | | | | | | |
| --- | --- | --- | --- | --- | --- | --- | --- | --- | --- | --- |
|  |  | Fever | Pain & swelling | Diarrhoea | Anaphylaxis | Cold & cough | Rash & itch | Nausea & vomiting | Others | Please specify (if other) |
| Day-1 |  |  |  |  |  |  |  |  |  |  |
| Day-2 |  |  |  |  |  |  |  |  |  |  |
| Day-3 |  |  |  |  |  |  |  |  |  |  |
| Day-4 |  |  |  |  |  |  |  |  |  |  |
| Day-5 |  |  |  |  |  |  |  |  |  |  |
| Day-6 |  |  |  |  |  |  |  |  |  |  |
| Day-7 |  |  |  |  |  |  |  |  |  |  |
